# A genome-wide ordered-subset linkage analysis for rheumatoid arthritis

**DOI:** 10.1186/1753-6561-1-s1-s101

**Published:** 2007-12-18

**Authors:** Desh Deep Mandhyan, Xana Kim-Howard, Matthew Gaines, Swapan K Nath

**Affiliations:** 1 Genetic Epidemiology Unit, Arthritis and Immunology Research Program, Oklahoma Medical Research Foundation, 825 Northeast 13th Street, Oklahoma City, Oklahoma 73104, USA

## Abstract

Rheumatoid arthritis (RA) is a chronic, complex autoimmune inflammatory disorder with poorly known etiology. Approximately 1% of the adult population is afflicted with RA. Linkage analysis of RA can be complicated by the presence of phenotypic and genetic heterogeneity. It is shown that the ordered-subset analysis (OSA) technique reduces heterogeneity, increases statistical power for detecting linkage and helps to define the most informative data set for follow-up analysis. We applied OSA to the family data from the North American Rheumatoid Arthritis Consortium study as part of the Genetic Analysis Workshop 15 (GAW15). We have incorporated two continuous covariates, 'age of onset' and 'anti-CCP level' (anti-cyclic citrinullated peptide), into our genome-wide ordered-subset linkage analysis using 809 Illumina SNP markers in 5713 individuals from 606 Caucasian RA families. A statistically significant increase in nonparametric linkage (NPL) scores was observed with covariate 'age of onset' in chromosomes 4 (*p *= 0.000003) and 9 (*p *= 0.002). With the covariate 'anti-CCP level', statistically significant increases in NPL scores were observed in chromosomes 2 (*p *= 0.0001), 18 (*p *= 0.00007), and 19 (*p *= 0.0003). Once we identified the linked genomic region, we then attempted to identify the best plausible parametric model at that linked locus. Our results show significant improvement in evidence for linkage and demonstrate that OSA is a useful technique to detect linkage under heterogeneity.

## Background

Rheumatoid arthritis (RA) is a chronic autoimmune inflammatory disorder of unknown etiology. Although environmental influences may trigger a response leading to the development of this autoimmune disease, both genetic and environmental factors are implicated in its pathogenesis [1]. It affects approximately 1% of the adult population with a female:male ratio ranging from 2:1 to 4:1 [2]. RA typically has an onset of symmetric joint swelling and reaches a peak incidence in the fourth and fifth decades of life [2]. RA-induced inflammatory response in the synovial membrane is typically chronic and destructive [3]. The main presenting symptoms of RA are pain, marked morning stiffness, impaired physical function, swelling, and tenderness of the joints. Constitutional symptoms of RA are fever, weight loss, and fatigue.

RA is a clinically heterogeneous disease and most likely has complex genetic involvement. The presence of underlying genetic heterogeneity of a trait often masks the effect of genetic markers with disease predisposing variants; hence, there may not be linkage in families in which the marker is not involved in the disease etiology [4]. One method used to address genetic heterogeneity and strengthen linkage findings is to incorporate phenotypic subsetting of the data [5]. Most phenotypic stratification approaches require that subsets be identified before linkage studies. We have applied this technique to detect linkage in another autoimmune disease, systemic lupus erythematosus (SLE) [6]. Alternatively, one can account for disease heterogeneity is by incorporating trait-related covariate data. Therefore, to map genes for complex trait, genetic analysis methods should acknowledge the presence of genetic heterogeneity when appropriate. In the present analysis, we used ordered-subset analysis (OSA), a powerful technique for linkage analysis of traits characterized by genetic heterogeneity [7]. In OSA, using different covariates based on clinical features of the phenotype or on environmental exposures, one can identify more homogeneous subsets of families. Linkage that would otherwise be missed may then be apparent. Therefore, the goal of OSA is to identify regions with increased linkage in a subset of families. Additionally, by increasing genetic homogeneity, OSA can also reduce the linkage interval as exemplified by other complex, diseases including Alzheimer disease [8].

The aims of our present analysis are to: 1) identify homogeneous subset of families and assess linkage and its location, 2) rigorously analyze the homogenous subsets of families with statistically significant chromosomal locations to find a parsimonious genetic model.

## Data and methods

We analyzed data from the North American Rheumatoid Arthritis Consortium (NARAC) study as part of the Genetic Analysis Workshop 15 (GAW15). Only Caucasian families were used for these analyses. Initially, out of the original 637 families, 31 families were removed due to mixed ethnicity or because they were uninformative for linkage analysis (single affected member per family). In larger families, ungenotyped individuals were trimmed to facilitate computation that otherwise was not possible due to time and memory constraints on the computer hardware used. We performed genome-wide linkage analysis of 809 Illumina SNP markers in 5713 individuals from 606 Caucasian rheumatoid arthritis families. Analyses were performed using FLOSS (Flexible Ordered Subset Analysis), MERLIN, GeneHunter, GeneHunter-Modscore, and Genehunter-Plus with the ASM (allele sharing model) module. We used several complementary programs to compare the accuracy of our results.

To date, several clinical and epidemiological factors have been identified as potential trait-related covariates for RA. Among them, increasing age of onset has been associated with worse outcome in RA, with evidence that there has recently been a shift towards an older age of onset [9]. There are also age differences in the strength of the association with risk factors like HLA, which might suggest that age has an effect on disease phenotype [10]. Recently, anti-CCP antibodies have been identified as highly specific for RA. These antibodies have also demonstrated prognostic utility with regard to radiographic outcomes [11,12]. Therefore, we selected covariates 'age of onset' and 'anti-CCP level' (anti-cyclic citrinullated peptide) and used them in OSA to identify homogeneous subgroup of families for linkage analysis. These covariates were used to assign linkage scores to each family using MERLIN. Mean covariate value for the family members was specified for each family and the families were ordered according to their covariate score. Multipoint linkage analysis was performed on all subsets of families with *k *smallest or *k *largest covariate scores. Thus, the subset type used here was extreme. The FLOSS program was used to create a covariate file for family covariate scores for all families and all covariates, and to calculate nonparametric linkage (NPL) scores. Permutation tests were used to assess the null hypothesis of independence of family linkage scores at each locus and family covariate scores. Each subset of homogeneous families that generated a statistically significant linkage was analyzed with GeneHunter to further confirm the NPL score.

Once we identified the linked genomic region, we then attempted to identify the most plausible parametric model (allele frequency, penetrance, and mode of inheritance) at that linked location. For each subset, parametric LOD scores were maximized using GeneHunter-Modscore. These allele frequencies and penetrance values were utilized in GeneHunter/GeneHunter-Plus with ASM module, which provides NPL, nonparametric LOD, parametric LOD, and heterogeneity LOD (HLOD) scores. In addition, information content was provided, which gave an index of the inheritance information extracted at each point in the genome by the marker genotyped. The LIN function (linear model to evaluate the evidence for linkage as defined by Kong and Cox [18]) of allele sharing method was used to calculate nonparametric LOD scores.

## Results

**Figure 1:**
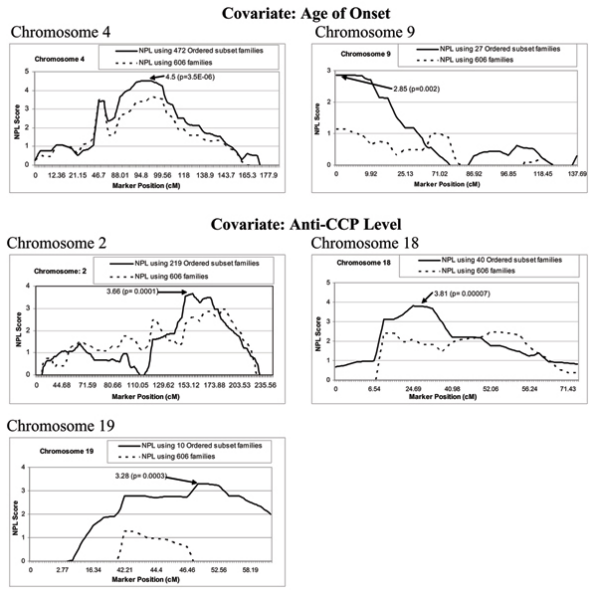
**Results of NPL analysis across the SNP marker positions**. Results of NPL analysis across the SNP marker positions in chromosomes (2, 4, 9, 18, and 19) showing the evidence of linkage in ordered subset of the families (solid line) based on covariate scores compared to all 606 families (dashed line) using GeneHunter.

The results of OSA along with the other relevant statistics are provided in Table 1. A significant increase in the evidence of linkage was observed at five chromosomal regions (Fig. 1). Using the covariate 'age of onset', statistically significant evidence of linkage was observed at chromosomes 4 (NPL = 4.5, *p *= 0.000003, peak at 102.03 cM, 472 families) and suggestive evidence was observed at chromosome 9 (NPL = 2.85, *p *= 0.002, peak at 0.59 cM, 27 families). With covariate 'anti-CCP level', statistically significant evidence of linkage was identified at chromosome 18 (NPL = 3.81, *p *= 0.00007, peak at 26.29 cM, 40 families), and suggestive evidence of linkage was observed at chromosome 2 (NPL = 3.66, *p *= 0.0001 peak at 154.11 cM, 219 families) and chromosome 19 (NPL = 3.28, *p *= 0.0003, peak at 52.21 cM, 10 families). The information content extracted at the linked region ranged from 46% to 80%.

**Table 1:** Summary of ordered subset linkage analysis

			Max				No. families		Covariate range			
							
Covariates	Chr. no.	Linkage peak (cM)^a^	New^b^	Old^c^	Delta^d^	*p*-Value (Delta Max)^e^	With defined covariate values	In best ordered subset^f^	Best^g^	Total^h^	*p*-Value (Permutations)	95% CI
Age of onset	4	102.03	4.45	3.55	0.9	0.012	606	+472	[31.5–83.0]	[11.0–83.0]	0.021	[0.0078, 0.0194]
	9	0.59	2.85	1.18	1.67	0.022	606	+27	[59.5–83.0]	[11.0–83.0]	0.022	[0.0141, 0.0349]
												
Anti-CCP level	2	154.17	3.72	2.83	0.9	0.048	590	+219	[133–413]	[0.8–413]	-0.951	[0.0302, 0.0742]
	18	26.29	3.73	2.46	1.27	0.022	590	+40	[234–413]	[0.8–413]	0.022	[0.0138, 0.0342]
	19	52.22	3.27	1.01	2.26	0.009	590	-10	[0.8–3.50]	[0.8–413]	0.009	[0.0057, 0.0142]

^a ^Location of maximum score, maximized over all ordered subsets.

^b ^Maximum score, maximized over all loci and ordered subsets.

^c ^Maximum score using all families with a covariate value, maximized over all loci.

^d ^Delta Max = (New Max) - (Old Max)

^e ^Probability of observing a change in LOD score greater than or equal to delta max if family linkage scores and family covariate scores are independent.

^f ^+ or -, the best ordered subset consists of families with the highest (+) or lowest (-) covariate scores.

^g ^Range of covariate values in the ordered subset with highest linkage score.

^h ^Range of covariate scores in the set of all families.

For each linkage region, the NPL score was significantly increased (*p *< 0.05) when we used all families versus subset of families. Further, the results with the GeneHunter program using the ordered subset families produced a statistically significant linkage that confirms the nearly identical NPL score obtained by the FLOSS program. Table 2 shows the results for parametric and nonparametric LOD scores obtained by incorporating the allele frequencies and penetrance of the best fitted model into GeneHunter-Plus with ASM module. Interestingly, these LOD scores are very similar at each linkage peak.

**Table 2:** Parametric and Non-parametric linkage analysis under the best fitted model

				Penetrance^a^									
											
Covariate	Chr. no.	No. ordered subset families	Disease allele frequency	f1	f2	f3	Position (cM) of peak MOD score	Peak MOD score	HLOD (α)	NPL score	*p*-Value	Position of peak MOD for ASM	Nonparametric LOD
Age of Onset	4	472	0.19	0.01	0.015	0.1	46.74	6.1	6.14 (0.89)	4.56	0.000002	96.27	6.03
	9	27	0.03	0	0.08	1	6.6	3.41	3.41 (0.99)	2.85	0.002	4.93	2.91
													
Anti-CCP level	2	219	0.18	0.01	0.015	0.1	151.68	4.09	4.08 (0.96)	3.72	0.0001	156.45	4.03
	18	40	0.13	0	0.99	1	24.69	5.44	5.44 (0.99)	3.81	0.00008	24.69	5.03
	19	10	0.07	0	0	0.04	52	3.32	3.32 (0.99)	3.28	0.0003	51.13	2.29

^a^f1, penetrance of wild-type homozygous; f2, penetrance of heterozygous; f3, penetrance of mutant homozygous

With the covariate 'age of onset', the age range in the ordered subset of families on chromosome 4 is between 31.5 and 83 years, whereas on chromosome 9 it is shifted more toward old age (59.5 and 83 years). The optimal range of 'anti-CCP level' in the ordered subset of families was greater in chromosomes 2 (133 to 413) and 18 (234 to 413), but lower for chromosome 19 (0.800 to 3.50). The values of peak maximized LOD (MOD) score and HLOD scores are nearly equal (which is expected). After using the allele-sharing model, not much difference was seen between the peak MOD score and nonparametric LOD scores produced by ASM except on chromosome 19.

## Discussion

We have identified five linked chromosomal regions (2, 4, 9, 18, and 19) that may harbor the susceptibility genes for RA. Previous studies [13,14] had identified linkage at chromosomes 2, 4, and 18. Our results also support the possibility of RA susceptibility gene in chromosomes 4 and 9 using the covariate 'age of onset' and in chromosomes 2, 18, and 19 using the covariate 'anti-CCP level'. It is interesting to note that the optimal range changes very little for 'anti-CCP level' on chromosome 2 and 18 linkages, but is quite different for chromosome 19, with absolutely no overlap. This would suggest an easily identifiable subset of family. However, we have only 10 families in this group, therefore, another independent replication is required to assess the validity of this finding.

We considered both nonparametric and parametric linkage analysis in this study. Both parametric and nonparametric results are very similar in terms of detecting the peak linkage locations. If we use the nonparametric LOD score then we have evidence for three statistically significant linkages at chromosomes 2, 4, and 18 that exceed the Lander and Kruglyak criteria (LOD score of 3.3) [15]. However, this threshold is not corrected for multiple testing (at least four different tests were performed: two different covariates and two different linkage methods, nonparametric as well as parametric). To maintain the overall genome-wide significance level (5% level), we have used an *ad hoc *correction procedure that raised the threshold of LOD score to 3.9. [This is calculated as: LOD_(corrected) _= LOD_(conv) _+ log_10_(#test) [16,17], where LOD_(conv) _is conventional LOD score to be significant = 3.3.] Interestingly, all three linkages remain significant after correcting for multiple testing.

For a complex trait like RA, successful identification of genetic risk loci has relied on the ability to minimize disease and genetic heterogeneity to increase the power to detect linkage. One way to account for disease heterogeneity is by incorporating covariate data. Phenotypically similar families may be genetically more homogeneous as well, in which case OSA can greatly improve the power of linkage analysis. Our results clearly show that 'age at onset' and 'anti-CCP level' are potentially two clinical markers that can be useful to detect linkage for RA and that OSA is an important technique to identify the linkage in the presence of heterogeneity. Such linkage studies could now be used for candidate gene as well as and fine mapping studies to identify the actual RA susceptibility genes.

## Conclusion

A genome-wide OSA was performed to identify the linkage for RA. We used two continuous covariates, 'age of onset' and 'anti-CCP level' to identify a more homogeneous group. We have identified two statistically significant regions with evidence of linkage at chromosomes 4 and 18 and three regions with suggestive evidence of linkage at chromosomes 2, 9, and 19. Our results clearly demonstrated that OSA is a useful technique to detect linkage under heterogeneity.

## Competing interests

The author(s) declare that they have no competing interests.

## References

[B1] Lekarski PM (2006). Genetics in rheumatoid arthritis (RA).

[B2] Grossman JM, Brahn E (1997). Rheumatoid arthritis: current clinical and research directions. J Womens Health.

[B3] Weyand CM, Goronzy JJ (1997). Pathogenesis of rheumatoid arthritis. Med Clin North Am.

[B4] Browning BL (2006). FLOSS: flexible ordered subset analysis for linkage mapping of complex traits. Bioinformatics.

[B5] Leal SM, Ott J (2000). Effects of stratification in the analysis of affected-sib-pair data: benefits and costs. Am J Hum Genet.

[B6] Namjou B, Nath SK, Kilpatrick J, Kelly JA, Reid J, James JA, Harley JB (2002). Stratification of pedigrees multiplex for systemic lupus erythematosus and for self-reported rheumatoid arthritis detects a systemic lupus erythematosus susceptibility gene (*SLER1*) at 5p15.3. Arthritis Rheum.

[B7] Hauser ER, Watanabe RM, Duren L, Bass MP, Langfield CD, Boehnke M (2004). Ordered subset analysis in genetic linkage mapping of complex traits. Genet Epidemiol.

[B8] Scott WK, Hauser ER, Schmechel DE, Welsh-Bohmer KA, Small GW, Roses AD, Saunders AM, Gilbert JR, Vance JM, Haines JL, Pericak-Vance MA (2003). Ordered-subsets linkage analysis detects novel Alzheimer disease loci on chromosomes 2q34 and 15q22. Am J Hum Genet.

[B9] Wiles N, Symmons DP, Harrison B, Barrett E, Barrett JH, Scott DG, Silman AJ (1999). Estimating the incidence of rheumatoid arthritis: trying to hit a moving target?. Arthr Rheumatol.

[B10] MacGregor A, Ollier W, Thomson W, Jawaheer D, Silman A (1995). HLA-DRB1*0401/0404 genotype and rheumatoid arthritis: increased association in men, young age at onset, and disease severity. J Rheumatol.

[B11] Bizzaro N, Mazzanti G, Tonutti E, Villalta D, Tozzoli R (2001). Diagnostic accuracy of the anti-citrulline antibody assay for rheumatoid arthritis. Clin Chem.

[B12] Meyer O, Labarre C, Dougados M, Goupille P, Cantagrel A, Dubois A, Nicaise-Roland P, Sibilia J, Combe B (2003). Anticitrullinated protein/peptide antibody assays in early rheumatoid arthritis for predicting five year radiographic damage. Ann Rheum Dis.

[B13] Amos CI, Chen WV, Lee A, Li W, Kern M, Lundsten R, Batliwalla F, Wener M, Remmers, Kastner DA, Criswell LA, Seldin MF, Gregersen PK (2006). High-density SNP analysis of 642 Caucasian families with rheumatoid arthritis identifies two new linkage regions on 11p12 and 2q33. Genes Immun.

[B14] Criswell LA, Gregersen PK (2005). Current understanding of the genetic aetiology of rheumatoid arthritis and likely future developments. Rheumatology.

[B15] Lander E, Kruglyak L (1995). Genetic dissection of complex traits: guidelines for interpreting and reporting linkage results. Nat Genet.

[B16] Kidd KK, Ott J (1984). Power and sample size in linkage studies. Cytogenet Cell Genet.

[B17] Ott J (1999). Analysis of Human Genetic Linkage.

[B18] Kong A, Cox NJ (1997). Allele sharing models: LOD scores and accurate linkage tests. Am J Hum Genet.

